# Stabilization of global temperature at 1.5°C and 2.0°C: implications for coastal areas

**DOI:** 10.1098/rsta.2016.0448

**Published:** 2018-04-02

**Authors:** Robert J. Nicholls, Sally Brown, Philip Goodwin, Thomas Wahl, Jason Lowe, Martin Solan, Jasmin A. Godbold, Ivan D. Haigh, Daniel Lincke, Jochen Hinkel, Claudia Wolff, Jan-Ludolf Merkens

**Affiliations:** 1 Faculty of Engineering and the Environment, University of Southampton, Highfield, Southampton SO17 1BJ, UK; 2 Biological Sciences, University of Southampton, Highfield, Southampton SO17 1BJ, UK; 3 Ocean and Earth Science, National Oceanography Centre Southampton, University of Southampton, Waterfront Campus, European Way, Southampton SO14 3ZH, UK; 4 Civil, Environmental, and Construction Engineering and National Center for Integrated Coastal Research, University of Central Florida, 12800 Pegasus Drive, Orlando, FL 32816-2450, USA; 5 Reading Unit, Met Office Hadley Centre, University of Reading, Reading, UK; 6 Priestley International Centre for Climate, University of Leeds, Leeds, UK; 7 Global Climate Forum, Neue Promenade 6, 10178 Berlin, Germany; 8 Geographisches Institut, Christian-Albrechts-Universität zu Kiel, Ludewig-Meyn-Strasse 14, 24098 Kiel, Germany

**Keywords:** sea-level rise, ocean pH, climate mitigation, climate adaptation, coastal impacts

## Abstract

The effectiveness of stringent climate stabilization scenarios for coastal areas in terms of reduction of impacts/adaptation needs and wider policy implications has received little attention. Here we use the Warming Acidification and Sea Level Projector Earth systems model to calculate large ensembles of global sea-level rise (SLR) and ocean pH projections to 2300 for 1.5°C and 2.0°C stabilization scenarios, and a reference unmitigated RCP8.5 scenario. The potential consequences of these projections are then considered for global coastal flooding, small islands, deltas, coastal cities and coastal ecology. Under both stabilization scenarios, global mean ocean pH (and temperature) stabilize within a century. This implies significant ecosystem impacts are avoided, but detailed quantification is lacking, reflecting scientific uncertainty. By contrast, SLR is only slowed and continues to 2300 (and beyond). Hence, while coastal impacts due to SLR are reduced significantly by climate stabilization, especially after 2100, potential impacts continue to grow for centuries. SLR in 2300 under both stabilization scenarios exceeds unmitigated SLR in 2100. Therefore, adaptation remains essential in densely populated and economically important coastal areas under climate stabilization. Given the multiple adaptation steps that this will require, an adaptation pathways approach has merits for coastal areas.

This article is part of the theme issue ‘The Paris Agreement: understanding the physical and social challenges for a warming world of 1.5°C above pre-industrial levels’.

## 1. Introduction

People and economic activity concentrate in coastal areas [1,2], which also contain vital environmental assets like saltmarshes, mangroves and coral reefs that underpin multiple ecosystem services. Hence, the impacts of climate change, including sea-level rise (SLR) and declining ocean pH, are a major threat to coastal zones. These are both being observed and are expected to continue, but the magnitude of effects can vary regionally [3–5].

Climate change mitigation comprises actions to limit the magnitude or rate of long-term climate change, usually by reduced human emissions of greenhouse gases. Several studies have analysed climate mitigation for coastal areas [6–8]. Many climate change factors respond directly to mitigation at a similar timescale to global temperature stabilization. However, for sea level, there is a long time delay in response and while the rate of SLR slows under mitigation, sea levels still continue to rise for centuries.

The Paris Agreement committed signatories to ‘Holding the increase in the global average temperature to well below 2°C above pre-industrial levels and to pursue efforts to limit the temperature increase to 1.5°C above pre-industrial levels, recognizing that this would significantly reduce the risks and impacts of climate change’ [9]. While sea level is not explicitly considered, it was an important background factor in discussions about the Agreement which was strongly driven by small island developing states (SIDS) who feel especially threatened by this aspect of climate change [10].

Hence this paper has two aims (i) to analyse the potential changes in coastal areas, including impacts and adaptation needs, under stringent stabilization targets relative to an unmitigated scenario and (ii) to consider the implications of such stabilization for the future of coastal areas, including adaptation and management policy. This includes a brief consideration of ecological effects that are anticipated for coastal systems under climate change and climate stabilization. The focus is on sea level and pH as two relevant climate parameters. Given the long timescale of SLR, we consider a number of time periods out to 2300 and examine and contrast emission scenarios leading to temperature stabilization at 1.5°C and 2.0°C with an unmitigated emission scenario. This timeframe is much longer than traditional analyses, which usually stop in 2100, and is necessary to see the full implications of climate change on coastal areas. Potential impacts are illustrated using appropriate indicators, as explained. These indicators should not be taken as projections.

Projections of temperature, sea level and ocean pH are developed with the Warming Acidification and Sea Level Projector (WASP) Earth system model for stabilization and unmitigated emissions [11,12]. They each comprise a large set of ensemble projections. Changes in ocean pH are less studied than SLR and temperature: with limited adaptation options available, reduction in CO_2_ emissions is currently viewed as the main practical way to address its impacts, although there are potential geoengineering options [13]. Undesirable effects of ocean acidification and warming may also be reduced indirectly by management of other anthropogenic stressors such as pollution drivers that interact adversely with climate drivers [14]. By contrast, many direct adaptation options are available for SLR (e.g. flood defences, flood-proof buildings, setbacks for new construction and restoring coastal ecosystems and geomorphological processes).

The paper is structured as follows. First, the methods to generate climate change scenarios are applied to the stabilization and unmitigated cases, and the resulting projections of SLR and ocean pH are analysed (§2). Second, the corresponding consequences of SLR under the same scenarios are analysed for global flooding and for selected vulnerable hotspots: small islands, deltas and coastal cities (§3). Third, we consider the coastal ecological effects of stabilized and unmitigated climate change (§4). Finally, the implications of these results are discussed, including climate and coastal policy implications (§5).

## 2. Projections of sea-level rise and ocean pH under 1.5°C and 2.0°C stabilization scenarios

A warming of global surface temperatures leads directly to global mean SLR from two main processes: (i) ice melt—the cryosphere adds additional water to the ocean---and (ii) thermosteric changes—warming of ocean waters leads to thermal expansion. Both these processes will continue for many centuries after a rise and stabilization of surface air temperatures due to the long timescale of cryospheric adjustment to elevated air temperatures (especially the large ice sheets), and the long timescale of the deep ocean temperature warming to surface warming [15]. This is often referred to as the ‘commitment to SLR’ [15,16]. Additional changes in global sea level are caused by anthropogenic effects that are not directly related to surface temperatures, such as changes in global land–water storage [17].

To project global mean SLR and ocean pH, the WASP Earth system model [11,12] is used to produce a large ensemble of 9 × 10^4^ simulations (see the electronic supplementary material), configured to be in good agreement with the range of projections of global mean surface warming and SLR from the Climate Model Intercomparison Project phase 5 (CMIP5) ensemble [12] following the SimHist ensemble therein. While the projection ranges of warming, SLR and the thermal expansion contribution to SLR are in close agreement between the WASP and CMIP5 ensembles across scenarios ranging from high-end RCP8.5 to significantly mitigated RCP2.6 [12], the WASP model is computationally efficient. Here, we use this computational efficiency to produce new ensemble projections for scenarios representing climate stabilization at politically agreed targets and compare these climate stabilization levels to an unmitigated scenario.

**Figure 1.**
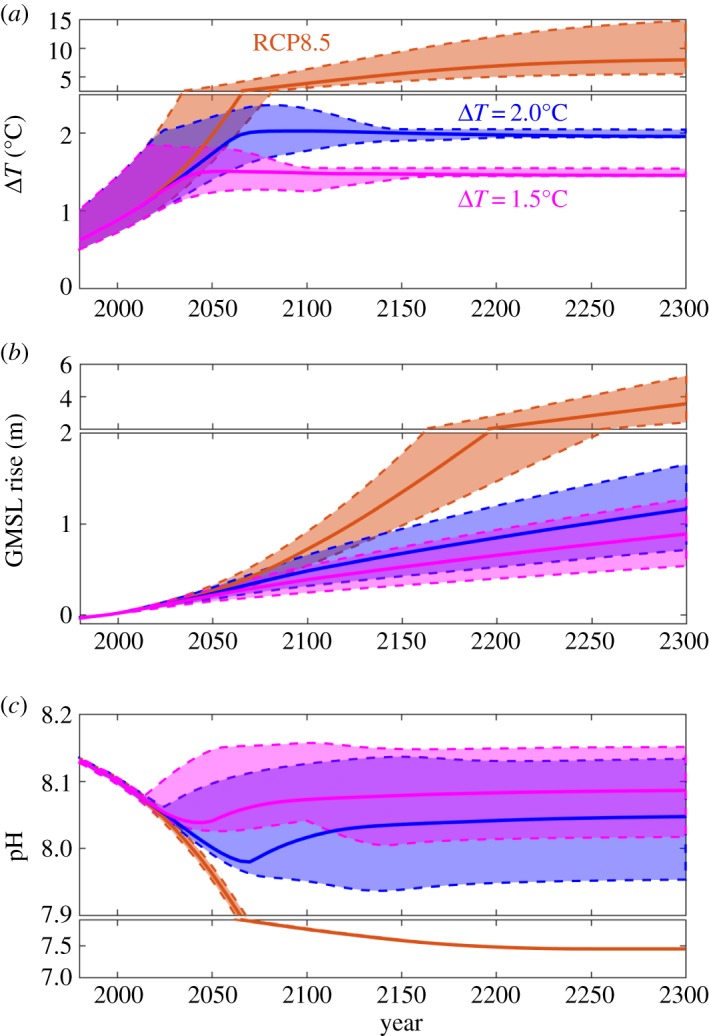
Future temperature rise, sea level and surface ocean pH projections to 2300 for a large (9 × 10^4^) ensemble using the WASP Earth system model. (*a*) Global temperature anomaly relative to pre-industrial, Δ*T* (°C), (*b*) global mean sea-level (GMSL) rise relative to 1986–2005 (m) and (*c*) surface ocean pH. The median ensemble projections over time (lines) and the 90% ranges within the ensemble simulations (shaded areas, from the 5th to 95th percentiles) are shown for RCP8.5 (red) and 2.0°C (blue) and 1.5°C (grey) stabilization scenarios.

Three future scenarios are considered (as described in the electronic supplementary material): (i) RCP8.5 [18] representing unmitigated emission under business as usual (figure 1, red), (ii) stabilization at 2.0°C warming (figure 1, blue) and (iii) stabilization at 1.5°C warming (figure 1, grey), consistent with the Paris Agreement (figure 1*a*).

The WASP ensemble projected trajectories of surface warming, SLR and surface ocean pH are shown in figure 1 for the three scenarios with key results listed in table 1. The uncertainty in future warming projections for RCP8.5 (figure 1*a*, red) reflects the uncertainty in the equilibrium climate sensitivity and uncertainty in the transient response of the climate system, with the WASP ensemble showing similar projection ranges to the CMIP5 projections [12]. For the 1.5°C and 2.0°C stabilization scenarios (figure 1*a*, grey and blue), the uncertainty in warming prior to 2100 reflects the uncertainty in how quickly the stabilization targets will be reached. After 2100, the uncertainty in warming decreases as the carbon emissions in the numerical experiments are tuned in each ensemble member to ensure that the stabilization target warming is followed. The stabilization scenarios both reach their target warming levels, while the high-end RCP8.5 continues to warm past 2100 due to significant ongoing emissions, reaching 5.5°C–14.8°C warming above pre-industrial by 2300 (table 1). It should be noted that while the warming and SLR projections from the WASP ensembles are historically tuned to give similar ranges to the CMIP5 ensemble-based AR5 projections (see electronic supplementary material and [12]), there are additional uncertainties not included within our projections (figure 1). In particular, historically unprecedented processes are not considered. This could include sudden future ice-sheet collapse or future warming exceeding expectations due to nonlinear feedbacks, such as accelerated methane release from warming permafrost. If these processes occurred, higher rises in sea level would be a likely consequence.

**Table 1.** Summary results of the WASP Earth system model for global mean temperature, global mean SLR and ocean pH and the two stabilization scenarios (1.5°C and 2.0°C) and the reference unmitigated (RCP8.5) emissions scenario. Results include the ensemble mean ± s.d. and the 90% range (5th to 95th percentiles) in parentheses.

	global mean temperature (relative to pre-industrial) (°C)			SLR (relative to 1986–2005 average) (m)			ocean pH		
time	1.5°C	2.0°C	RCP8.5	1.5°C	2.0°C	RCP8.5	1.5°C	2.0°C	RCP8.5
1986–2005	0.8 ± 0.2			0.0			8.11 ± 0.00		
	(0.7–1.3)						(8.10–8.11)		
2050	1.5 ± 0.2	1.8 ± 0.3	2.1 ± 0.5	0.21 ± 0.04	0.23 ± 0.04	0.26 ± 0.04	8.06 ± 0.04	8.01 ± 0.04	7.96 ± 0.01
	(1.2–1.8)	(1.4–2.2)	(1.6–3.2)	(0.14–0.28)	(0.17–0.30)	(0.19–0.32)	(8.03–8.15)	(7.97–8.11)	(7.95–7.97)
2100	1.5 ± 0.1	2.0 ± 0.2	4.1 ± 1.0	0.39 ± 0.09	0.49 ± 0.10	0.72 ± 0.11	8.08 ± 0.04	8.02 ± 0.06	7.75 ± 0.01
	(1.2–1.6)	(1.8–2.3)	(3.0–6.3)	(0.24–0.54)	(0.31–0.65)	(0.54–0.91)	(8.04–8.16)	(7.95–8.13)	(7.74–7.76)
2300	1.5 ± 0.1	2.0 ± 0.1	8.8 ± 3.1	0.89 ± 0.23	1.17 ± 0.29	3.65 ± 0.89	8.09 ± 0.04	8.04 ± 0.06	7.45 ± 0.00
	(1.4–1.5)	(1.9–2.0)	(5.5–14.8)	(0.53–1.27)	(0.71–1.65)	(2.40–5.27)	(8.02–8.15)	(7.95–8.13)	(7.74–7.75)

The range of future SLR projections in the WASP ensemble of simulations (figure 1*b*) reflects the uncertainty in the cryospheric response to future warming, uncertainty in the ocean heat uptake response to future warming and uncertainty in the simulated future warming itself. For the stabilization scenarios, some simulations see an overshoot of the warming target during the twenty-first century while others never exceed the stabilization (figure 1*a*, grey and blue shaded). This temperature history has an impact on the SLR projections: projected SLR at year 2100 is correlated with simulated warming at 2035 (which is when the maximum range of temperatures occurs across the ensemble before stabilization later in the twenty-first century) with a coefficient of determination (*R*^2^) of 0.19: the simulations with the larger warming overshoot above 1.5°C in 2035 tend to have larger SLR at 2100 than those with less overshoot (figure 1*a,b* grey). This is consistent with SLR being more related to the time integral of warming than the instantaneous warming at any particular time. However, the majority of the variation in the ensemble SLR projections for a specific warming target arises from variation in the model sensitivities of SLR to warming. This suggests that more SLR should be expected for scenarios with warming overshoot, but that the effect is less than the total uncertainty in SLR due to the sensitivities of the cryosphere and ocean heat uptake to surface warming. For RCP8.5, the WASP ensemble ranges agree well with the CMIP5 projections [12].

For the 1.5°C and 2.0°C scenarios, SLR continues despite the ensemble-median temperature stabilizing in the mid-twenty-first century. By 2100, projected SLR is 0.39 ± 0.09 m and 0.49 ± 0.10 m under 1.5°C and 2.0°C stabilization, respectively. There is a large overlap between the two stabilization scenarios, indicating a lack of climate sensitivity due to the commitment to SLR. By 2300, SLR is projected at 0.89 ± 0.23 m and 1.17 ± 0.29 m for the 1.5°C and 2.0°C scenarios, respectively, and the benefits of stronger mitigation are more apparent. Compared with the unmitigated RCP8.5 scenario, median SLR in 2300 is reduced by 2.76 m (75%) and 2.48 m (68%) under 2.0°C and 1.5°C stabilization, respectively. Expressed as a linear rate of SLR from 2100 to 2300, the median changes are 2.5 mm yr^−1^, 3.4 mm yr^−1^ and 14.7 mm yr^−1^ under the three scenarios, respectively. At the 95th percentile, the RCP8.5 could exceed 20 mm yr^−1^, seven times faster than satellite observations of SLR during the early twenty-first century [3].

The range of projected changes in surface ocean pH in the WASP ensembles for the 1.5°C and 2.0°C scenarios reflects the uncertainty in the future atmospheric CO_2_ trajectory compatible with each stabilization target (figure 1*c*, grey and blue). For RCP8.5 there are prescribed CO_2_ concentrations resulting in limited range in terms of surface ocean pH (figure 1*c*, red). The pre-industrial surface ocean pH in the WASP model is 8.2, and this decreases to 8.1 by the early twenty-first century (figure 1*c*) as atmospheric CO_2_ increases and dissolves in the surface ocean as inorganic carbon. For stabilization at 1.5°C, projected surface ocean pH declines to 8.08 ± 0.04 in 2100 and to 8.09 ± 0.04 by 2300. For stabilization at 2.0°C, projected surface ocean pH declines to 8.02 ± 0.06 in 2100 and to 8.04 ± 0.06 in 2300. Under the RCP8.5, surface ocean pH declines to 7.75 ± 0.01 by 2100 and 7.45 by 2300. Hence, the benefits of stabilization in terms of reducing the surface ocean pH change are dramatic. Relative to RCP8.5, stabilization at 1.5°C and 2.0°C reduces the change in surface ocean pH from pre-industrial by 73% and 60% by year 2100, and by 85% and 79% by year 2300, respectively.

For the temperature stabilization pathways, the uncertainties in our projections of surface ocean pH and SLR increase with time. Our projections with WASP assume that climate sensitivity is constant in time. However, century-scale feedbacks can alter the climate sensitivity [19] and may affect the CO_2_ concentration and projected surface ocean pH for a given climate stabilization target beyond year 2100. Our SLR projections beyond year 2100 assume a smooth path towards the eventual equilibrium SLR for a given climate stabilization. However, some processes may occur relatively suddenly, such as ice-sheet collapse [3], and this adds additional uncertainty to SLR beyond year 2100 that is not encapsulated within the WASP model projections.

## 3. Impacts of sea-level rise under climate stabilization

SLR has a range of impacts on coastal areas, including (i) increased coastal flooding and inundation; (ii) coastal morphodynamic changes, especially erosion; (iii) ecosystem changes such as wetland change and loss; and (iv) hydrological and salinization effects in coastal surface and ground waters [20–22]. There is a complex interplay between these factors and morphodynamic and ecosystem change can influence coastal flooding, for example. In this analysis, we mainly focus on the potential of coastal flooding and inundation as the impacts could be dramatic [23]. Importantly, SLR is not occurring in isolation and multiple climates and non-climate drivers are shaping coastal areas [24], although the magnitude of these drivers varies significantly in space. Some key examples are population growth, urbanization, changing sediment supply and land uplift/subsidence, resulting from natural (i.e. glacial isostatic adjustment) and anthropogenic subsidence (due to ground fluid extraction) processes [25,26]. Hence, it is important to set SLR and climate change in this broader context. Coastal adaptation is also critical to consider as this can greatly reduce impacts [23,27,28], and protection already allows large populations to remain in locations that would otherwise be highly hazardous, such as the western Netherlands [29] and parts of China's coastal lowlands [30].

### (a) Global coastal flood impacts

To analyse the possible benefits of stabilization in terms of reduced coastal flooding, the three global mean SLR scenarios shown in figure 1 were assessed at a global scale using the Dynamic Interactive Vulnerability Assessment modelling framework (DIVA, v. model 2.0.1, database 32) (see electronic supplementary material). Storm characteristics are assumed constant and erosion is not considered—changes in both these factors may enhance the impacts presented. Expected number of people flooded per year is used as an impact indicator. To address uncertainty, the 5th, 50th and 95th percentiles of the sea-level projections were considered, together with the five shared socio-economic pathways, which reflect a range of future population and economic conditions to 2100 [31,32]. Beyond 2100, a stable population and population distribution are assumed, following the impact assessment community convention [33] and the results are indicative. Adaptation in the base year was represented by protection in the form of sea dikes, estimated following a demand-for-safety function where safety (and dike height) mainly vary with population density and wealth [23]. Dikes are initialized in 1995 with no subsequent upgrade so that the impacts (and adaptation needs) due to SLR are apparent.

**Figure 2.**
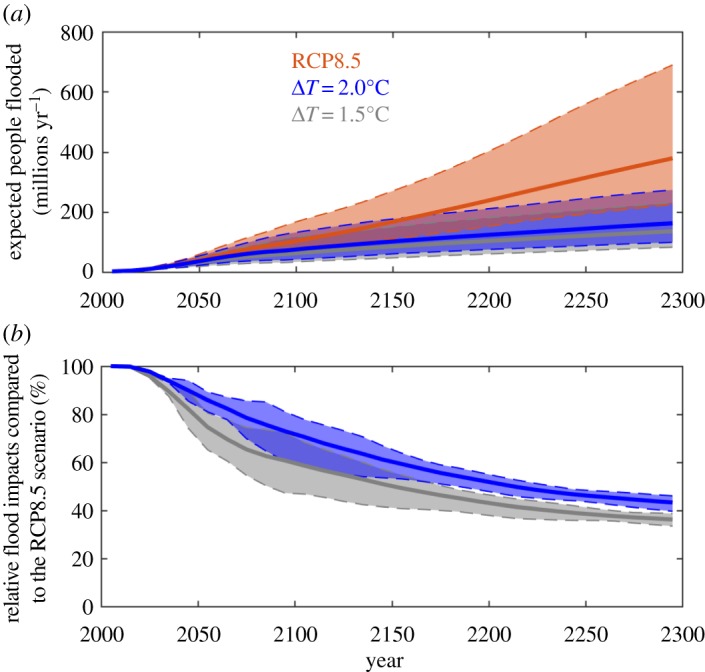
Climate stabilization and global coastal flooding. (*a*) Expected number of people flooded (millions yr^−1^) versus time from 2000 to 2300 for stabilization and unmitigated SLR scenarios across all socio-economic scenarios for each emissions pathway. (*b*) Relative comparison of impacts under stabilization, showing the percentage of impact, normalized by unmitigated (RCP8.5) impacts for the same socio-economic scenario. In both cases, this assumes no adaptation (i.e. dike upgrade) and the numbers are indicators, not projections. The mean ensemble projections over time (lines) and the range are shown for RCP8.5 (red) and 2.0°C (blue) and 1.5°C (grey) stabilization scenarios.

Figure 2*a* illustrates the expected number of people flooded annually around the coasts to 2300 under these assumptions. Absolute impacts grow with time under all scenarios, but this growth is slower under climate stabilization. Reduced impacts are apparent during the twenty-first century, and this reduction grows in the twenty-second and twenty-third centuries. Figure 2*b* shows that 1.5°C stabilization (grey) has a greater relative reduction in impacts than 2.0°C stabilization (blue). The range overlaps at first and separates in the later twenty-second century. The mean impacts relative to the unmitigated RCP8.5 scenario are 60% and 72% in 2100, and 36% and 43% in 2300, respectively. The absolute growth in impacts means that while stabilization avoids significant impacts, coastal risk still grows progressively under both climate stabilization scenarios to 2300. Hence, total risk can only be maintained at current levels with significant adaptation in addition to climate stabilization.

The effects on small islands, deltas and coastal cities are examined in more detail below.

### (b) Small island developing states

Globally, there are more than 50 SIDS and many more small islands which are highlighted to be at high risk from climate change in multiple Intergovernmental Panel on Climate Change (IPCC) reports (e.g. [34,35]). In the Paris Agreement, SIDS are mentioned on five occasions and noted as being ‘particularly vulnerable’ due to their ‘significant capacity constraints'. This is particularly acute as many islands are variously remote (e.g. Marshall Islands), geographically dispersed (e.g. Federated States of Micronesia), poorly developed (e.g. Haiti), lack a skills base and/or low lying (e.g. Maldives) [36].

Climate change will affect SIDS through multiple factors, including SLR, oceanic warming, changing cyclones and changes in precipitation and temperature patterns [35]. Higher sea levels and wind-driven water levels are anticipated to increase flooding and erosion, plus increase the likelihood of salinization of freshwater resources [37]. Low island nations such as the Maldives, Kiribati, Tuvalu and Turks and Caicos Islands are particularly threatened. Significant shoreline changes are observed on small islands, but it is difficult to attribute these to SLR (e.g. [38,39]). Even islands which are not low in elevation (e.g. Grenada, Seychelles, Fiji) are threatened as infrastructure is concentrated at low elevations close to the coast (e.g. [40]). Indeed, 50% of Caribbean and Pacific Islanders live within 1.5 km of the coast [41]. With many islands relying on tourism, sustaining this industry is a major concern.

Some scientists paint a bleak prospect for small islands under SLR, with the recent loss of entire islands in the Solomon Islands being considered indicative of their wider future [42]. Other scientists are more optimistic and consider that coral islands may grow with SLR [43–45]. For example, Kench *et al.* [46] mapped Pacific islands from 1897 to 2013 and found a net gain in land, despite SLR. This suggests that some islands may cope with slow SLR if left to accrete naturally. Such accretion can take various forms, including vertical accretion due to overtopping waves and/or horizontal accretion as new sediment welds to islands. Developed islands which are constrained by coastal defences may be less able to accrete. An example of this is Malé, Maldives (average height 1 m a.m.s.l. and surrounded by dead reef, limiting new coral-derived sediment supply). Here, SLR poses a serious threat when considered alongside energetic swell waves which periodically cause flooding [47]. The RCP8.5 SLR scenario threatens Malé this century, while SLR associated with 1.5°C and 2.0°C poses a significant threat by 2300.

Climate stabilization offers substantial benefit to small islands, buying time to adapt to SLR, including possible relocation, upgraded defence or even artificial raised island construction [48]. The reduction in coastal ecosystem impacts due to stabilization discussed in §4 offers substantial but unquantified benefits sustaining island livelihoods and the wider environment, including allowing natural accretion. SIDS need help and time to build the capacity to plan and finance adaptation. Migration is seen as the final adaptation option for many low-lying islands in the face of SLR [49] and longer-term planning is being considered by several island nations in the form of land purchases or other arrangements [49,50]. Migration in islands is already a widespread practice (e.g. Vunidogoloa, Fiji [51]; Guna, Panama, [52]; the Maldives [53]), reflecting multiple social, economic and developmental drivers. Planning for SLR and changing environmental conditions must be considered in the light of these broader issues.

### (c) Deltas

Unlike small islands, deltas are not specifically mentioned in the Paris Agreement, but they are also highly vulnerable to large floodplains containing much larger populations. Deltas in mid and low latitudes provide homes to about 500 million people worldwide [54] with a concentration in south, southeast and east Asia [55]. These deltas are experiencing rapid environmental change reflecting changes in the catchments, such as reduced sediment flux due to dams, and within the deltas themselves due to changes such as urbanization and flood defence. Deltas naturally subside due to sediment compaction and crustal loading, and this is widely enhanced by oxidation due to drainage and sub-surface fluid withdrawal. This subsidence adds significantly to relative SLR in deltas [56,57] with estimated present mean subsidence of 3.6 mm yr^−1^ across a sample of 46 major deltas worldwide, with a range of 22 mm yr^−1^ subsidence in the Indus Delta, Pakistan to land surface rise in the Krishna Delta, India reflecting sediment aggregation. As a result, significant land areas in many deltas are already below normal high tides and depend on defences and drainage to be habitable [58]. Therefore, deltaic areas are highly vulnerable to climate change and SLR [54,57]. Stabilizing sea level will not stop non-climate factors, and deltas will continue to change in a hypothetical stable climate, including experiencing relative SLR due to subsidence, as occurred in the Rhine Delta, The Netherlands over the last millennia [29].

Given the multiple interacting processes shaping deltas, it is difficult to diagnose the effect of a single driver such as climate-induced SLR, or climate change as a whole (e.g. climate-induced changes in run-off from the associated catchment). Further, the process of determining the relative importance of these effects can be frustrated by the moderating effects of human activity within the delta. Taking the Ganges–Brahmaputra Delta in Bangladesh as an example, these include changing upstream land use and catchment regulation (dams and barrages) [59,60]; extensive land cover change in the delta, including extensive polder systems which regulate hydrology and sediment flux and the creation of extensive shrimp farms; and regional and local subsidence [61], with over 1 m local loss of elevation in many polders due to drainage, oxidation and consolidation [62]. These, in turn, have had profound morphodynamic and hydrodynamic consequences such as reducing flow and encouraging channel siltation, which are only now being recognized (e.g. [63]). In comparison to observed climate-induced SLR to date, these anthropogenic influences dominate observed changes here and in many other populated deltas. An extreme example is the Nile where the sediment supply that produced the delta has been almost totally removed due to a large single dam on the main river (at Aswan) [64].

Electronic supplementary material, figure S1, shows the reduction in relative SLR to 2100 as a function of stabilization and subsidence. For a hypothetical no subsidence case, stabilization at 2.0°C and 1.5°C avoids 33% to 47% of the relative rise, respectively, while for a uniform subsidence of 6 mm yr^−1^, the reduction in relative SLR is only 18% to 25%. (Note that much higher rates of subsidence exceeding 10 mm yr^−1^ are observed in some coastal cities on deltas [65], but this is not considered here.)

As with small islands, climate stabilization will reduce impacts in deltas, but climate-induced SLR continues, and is compounded and maybe dwarfed by non-climate changes as listed above. Again long-term adaptation and planning need to be considered in deltas, with examples emerging such as the draft Bangladesh Delta Plan 2100 [66]. In the face of SLR and subsidence, delta management has three fundamental choices: (i) retreat; (ii) protect (e.g. replicate the strategy adopted by The Netherlands); or (iii) build elevation (with sedimentation). Innovative management regimes are being considered in terms of working with nature and promoting sedimentation in the Mississippi and Ganges–Brahmaputra deltas [67,68]. This approach is in its infancy, but provides deltas with a sustainable option against SLR and subsidence, assuming that upstream sediment supplies are sustained. Climate stabilization means that such adaptation strategies are more likely to be successful.

### (d) Coastal cities

Coastal urbanization has been a defining feature of the world's coast through the twentieth and early twenty-first century. In 2005, there were 136 coastal cities with more than 1 million people and a total population of 400 million people [69]. While coastal cities continue to grow in size and in number, there are expectations that unmitigated SLR will lead to their widespread abandonment (e.g. [70,71]), reinforced by widely reproduced media images [72]. However, based on empirical experience many cities have adapted to both extreme events and significant relative SLR (i.e. subsidence), and such adaptation seems feasible over the coming decades [27].

**Figure 3.**
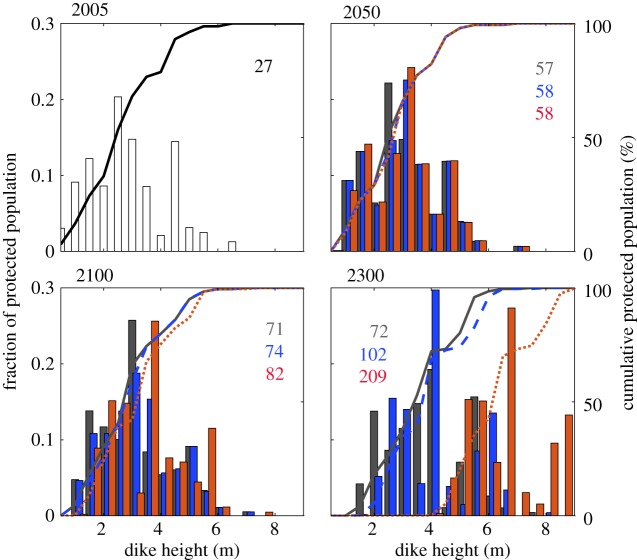
Estimates of the dike heights required to protect the major coastal cities under the three median climate scenarios from 2005 to 2300 (red: RCP8.5; blue: 2°C; grey: 1.5°C). The bars show the fraction of the floodplain population protected by the respective dike height while the lines show cumulative population. The numbers in the panels are cumulative number of people protected (in millions).

Here, we follow [27] and estimate required dike heights as an indicator of stabilization benefits across 136 coastal cities (see electronic supplementary material) for median SLR under our three scenarios. The projected dike heights only become distinct after 2100 (figure 3). By 2300, the dike heights under the unmitigated (RCP8.5) scenario (red) are more than 2 m higher than under the stabilization scenarios, with a commitment to further raising beyond 2300. Population in the floodplain increases through time, with the differences across scenarios being small to 2050 and larger thereafter. Hence, defence failure would affect larger numbers of people and increase impacts: this would become increasingly catastrophic with time, especially under the unmitigated scenario.

There is limited analysis of the implications of large magnitudes of SLR on individual coastal cities, with a few cities such as Amsterdam, New York and London being exceptions. The Thames Estuary 2100 (TE2100) project considers the adaptation options and related issues against up to 5 m of SLR [73,74]. It takes an explicit adaptation pathways approach. This recognizes that adaptation will be a process comprising a series of upgrades into the future, rather than a single decision, and the required magnitude of each upgrade is uncertain [75]. The focus of the TE2100 project was to 2100, but the analysis can be applied to post-2100 change. According to our analysis, by 2300 SLR of more than 2.5 m occurs for the RCP8.5 scenario across the 5th to 95th percentile range, which means that one viable protection option is available: a new downstream barrage (or a major retreat). By contrast, under both stabilization scenarios a much wider range of options are available to 2300. Hence, the availability of the TE2100 analysis informs adaptation for London beyond 2100, including for the SLR scenarios considered here.

## 4. Coastal ecological effects under climate stabilization

In contrast to the SLR effects already discussed, ecological effects of climate change are more complex to define because they reflect the interdependencies between multiple climate drivers, biotic and environmental context and anthropogenic activity [76]. Species must adapt to rising CO_2_ while simultaneously acclimatizing to shifts in environmental conditions, including sea surface temperature (SST) rise and SLR that operate over longer timescales, and exhibit considerable spatial variation [14,77–79]. In addition, ecosystems are open and may interact with adjacent ecosystems [80]. Such complexities are difficult to anticipate across the spectra of future scenarios and the severity and direction of ecosystem response can depend on timing and local context [14,38]. While climate-related impacts are already being detected in some coastal marine ecosystems (e.g. regime shifts [81]), understanding of key thresholds above natural variability in coastal marine ecosystems is limited [82]. Seagrasses, for example, when exposed to plausible near-future climatic conditions, exhibit higher rates of photosynthesis, carbon fixation and growth. Such growth enhancement, however, compromises the plants’ biomechanical properties, increasing long-term vulnerability to storm conditions and compromising protective functions [83]. Similarly, the response of a species to a particular ensemble of climatic conditions can depend on life stage or physiological condition, which are seldom assessed [84], or on uncertain changes in biotic interactions [85]. Hence, understanding ecological responses under climate change is a major challenge [86], not least because the linkages between the projections of SST rise, SLR, acidification and the biological community are insufficiently constrained. Most relevant experimental investigations only consider one or two climatic variables, although more sophisticated experimental designs are being developed [87]. Most importantly, the basic science about the effects of warming between 1.5°C and 2°C has received little attention [88,89]. These are not a linear function of temperature rise [14,90], precluding extrapolation and interpretation of the climate scenarios produced in this paper. Further, species and ecosystem responses integrate multiple drivers of change [37,91], increasing the risk of negative ecosystem responses [92]. Estimates of species and ecosystem vulnerability tend to focus on large-change scenarios such as RCP8.5 in 2100 and a limited range of drivers (e.g. [93–95]), rather than expected climate change in the coming decades [96] or anticipated changes in species composition, interaction and behaviour following acclimation and/or adaptation to novel circumstances [97].

Limited evidence is available, but several studies [95,98–100] suggest that large benefits will accrue for coastal ecosystems if global warming is stabilized at or below 2°C, although most available analyses exceed 1.5°C before 2100. As noted in §3b, atolls may be able to keep pace with SLR, but the interdependencies that exist between neighbouring habitats, such as coral reefs, seagrass and mangroves [80], mean that impacts are unlikely to be avoided, especially in low-lying areas [4]. While some species appear to be resilient to moderate levels of pH reduction and warming [101,102], the fate of others, such as reef-building corals, is not yet clear [78,103], but see [104,105]. Increases in temperature and precipitation extremes [106,107] are likely to be sufficient to cause functionally important shifts in habitat type (e.g. algal to coral dominated reefs [108]) and/or lead to local or regional mass mortality [109]. Similarly, SLR has the potential to increase flood frequency in tropical coastal habitats [110,111] and generate substantive saltmarsh degradation and/or loss if not compensated by accretion [46], while elevated atmospheric [CO_2_] may enhance plant growth [112] but impair soil microbial community structure [113]. As the interplay between these and other interacting factors, including those not related to a changing climate (e.g. [114]), can be important in determining ecosystem response, there remains considerable uncertainty in projecting the most likely coastal ecological effects under climate stabilization.

## 5. Discussion

The analysis presented here emphasizes the different timescales by which stringent climate mitigation leading to temperature stabilization affects coastal areas. Climate variables linked to temperature and greenhouse emissions, such as SST and ocean pH, can be stabilized over the timescale of about a century. Changes in other relevant climate variables for coasts, such as the characteristics of tropical and extratropical cyclones, which depend on surface and atmospheric temperature, are likely to behave similarly. Further pH and SST stabilization avoids significant, but not all, coastal ecosystem impacts. However, this is poorly quantified reflecting scientific uncertainty, including a lack of understanding of how different climate and non-climate drivers might interact.

By contrast, the timescale of SLR is much longer and while slowed, SLR continues for at least three centuries under both 1.5°C and 2.0°C stabilization. (A hypothetical global cooling would be required to stabilize sea level [115] more quickly.) For example, our median estimate of global SLR by year 2100 for RCP8.5 is 0.72 m, relative to the 1986–2005 average. This rise occurs 65 years later for stabilization at 2.0°C and 130 years later for stabilization at 1.5°C. Hence, many twenty-first century impacts are delayed rather than avoided with mitigation and SLR remains a long-term challenge under greenhouse gas and temperature stabilization.

The simulations in this paper project that large rises in sea level of up to 5 m by 2300 might occur under RCP8.5, which could be further increased by potential processes not included in our model such as rapid future collapse of the Greenland ice sheet. Such large SLR will fundamentally change the world's coast, eroding or submerging coastal areas, except for those areas where natural systems have sufficient sediment supplies to compensate for SLR and/or where humans choose to protect. While protection is technically feasible and likely to occur in many urban areas following current practice, provision of such adaptation raises questions of delivery, maintenance, governance and ultimately residual risk—the consequences of any failure would often be catastrophic, and could dwarf the consequences of recent disasters, such as Hurricane Katrina's impact on New Orleans [27]. Climate stabilization reduces these challenges and gives substantially more time to adapt to the SLR that does occur. This includes natural adjustments such as wetland and atoll accretion, as well as human adaptation.

This analysis reinforces the earlier IPCC conclusions that the best societal response to SLR is climate change mitigation to reduce SLR to manageable levels, and adaptation in response to the residual unavoidable rise [4,116]. As already noted, median global rises in sea level for 1.5°C and 2.0°C stabilization by 2300 are about 0.9 m and 1.2 m, respectively. Without adaptation, these changes are of concern to populated and economically important low-lying areas in SIDS explicitly mentioned in the Paris Agreement, as well as small islands in general, deltas and coastal cities as considered here. However, over these long timescales, adaptation is feasible and essential as SLR cannot be avoided. A stronger message of the need for long-term preparation and planning for SLR is required. The adaptation pathways approach, as exemplified by the TE2100 project for London, could be adapted and followed more widely around the world's developed coasts to prepare for this change including the uncertainties [75,117]. Such analysis allows recognition of when problems might emerge, what the potential solutions are, and how they might be integrated with wider plans for coastal development. Such adaptation will be a multi-step process over many decades and longer [118]. In locations society wishes to protect, the approach of building elevation as opposed to building dikes is worthy of more consideration, as this addresses the fundamental issue of growing residual risk. For example, Singapore will raise all new land claim to allow for SLR and other low-lying areas as redevelopment allows [119]. Urban areas in small islands require particular attention as their options under rising sea levels are most limited.

Our projections of SLR represent one model (tuned to emulate the AR5 projections deriving from the CMIP5 ensemble [12]), and hence are consistent with the AR5 SLR scenarios [20]. We note earlier studies [120,121] using semi-empirical models estimated higher rises in sea level by 2300 for 2°C stabilization. We also recognize the potential role of physical processes that have not yet been observed or that are not fully understood which might increase SLR projections, especially under the unmitigated case. As climate science develops so the approach used here can be repeated, re-evaluating our results.

One of the main benefits of lower long-term temperature goals is the reduced likelihood of irreversible deglaciation of the large ice sheets. Some authors suggest we may be close to or have even crossed such thresholds for the Greenland [122] and Antarctica ice sheets [123]. This highlights the need to continue investigations of SLR and its different components both with and without climate stabilization. This should be combined with monitoring of SLR.

The Paris Agreement [9] signalled a transformational political change in greatly limiting temperature rise. Does 1.5°C stabilization offer coastal areas distinct outcomes to 2.0°C stabilization? The range of resulting SLR scenarios still partially overlap in 2300, although 2.0°C stabilization has higher rises, and resulting impacts and adaptation needs. Understanding the difference between these two futures also requires consideration of the feasibility of adaptation for SLR and non-climate changes such as subsidence in deltas. Nonetheless, both climate stabilization scenarios show a large reduction in potential impacts as opposed to the unmitigated RCP8.5 scenario, and the avoided impacts grow with time. Hence, the results clearly show that even with stringent mitigation, society will also need ongoing adaptation to SLR in coastal areas to avoid significant damage.

## 6. Conclusion

This contribution has considered the effect and implications of stringent climate stabilization scenarios for coastal areas in terms of reduction of impacts/adaptation needs to 2300. This is a longer timescale than earlier analyses. Simulations of climate stabilization at 1.5°C and 2.0°C show that ocean pH and temperature stabilize within a century. This means that significant coastal ecosystem impacts are avoided, but detailed quantification is not possible with current understanding. More systematic scientific investigation of these changes would be useful. By contrast, SLR is only slowed compared to unmitigated emissions and continues to 2300 (and beyond). Hence, while coastal impacts due to SLR are reduced significantly by climate stabilization, especially after 2100, the potential impacts continue to grow for at least several centuries. Importantly, SLR under stabilization in 2300 exceeds unmitigated SLR in 2100, raising concerns for vulnerable areas such as small islands, deltas and coastal cities. Hence, consideration of adaptation to SLR remains essential under both the climate stabilization scenarios considered here. The best societal response to SLR is climate change mitigation to reduce the risk to manageable levels, and adaptation in response to the residual unavoidable rise. Given the long timescale of the issue, linking adaptation with wider coastal development planning has strong merits. As adaptation will involve multiple steps, exploration of adaptation pathways would be an appropriate approach to guide such planning.

The implications of climate mitigation leading to atmospheric temperature stabilization for SLR are in need of more recognition and assessment. The physics that lead to the commitment to SLR were reported in the first IPCC Assessment [124] and again in the third IPCC Assessment [125]. Then the impact and adaptation implications were discussed in the fourth and fifth IPCC Assessments [4,116]. Nonetheless, the climate policy process has continued to focus on temperature mitigation as if it was a universal solution to human-induced climate change. As this paper demonstrates in more detail than earlier assessments, for SLR this is not the case and the policy process needs to consider the implications of this fundamental physical constraint. While this paper has focused on 1.5°C and 2.0°C stabilization, this conclusion is true for SLR under any temperature stabilization scenario.

## Supplementary Material

Methods Used

## Supplementary Material

Data for Nicholls et al FINAL

## Data accessibility

The datasets supporting this article have been uploaded as part of the electronic supplementary material.
