# Supplementary material for: Stabilization of global temperature at 1.5°C and 2.0°C: implications for coastal areas
Source: Philos Trans A Math Phys Eng Sci. 2018 Apr 2;376(2119):20160448. doi: 10.1098/rsta.2016.0448 (PMC5897821; doi:10.1098/rsta.2016.0448)
Supplement: Methods Used [file rsta20160448supp1.doc]

**Methods**

**Warming Acidification and Sea-level Projector (WASP) Earth system model**

The Warming Acidification and Sea-level Projector (WASP) Earth system model is a computationally efficient 8-box representation of the Earth system that is used for large ensemble simulations with varying parameter values [1]. WASP uses a hybrid method for projecting global-mean sea-level rise, with a mechanistic representation of sea-level rise due to ocean thermal expansion (the thermosteric contribution) combined with a semi-empirical representation of sea-level rise due to ice melt (the ice-melt contribution).

Following the methodology of [1]; see SimHist ensemble therein) WASP is used to run 10-million simulations with different model-parameter value combinations from years 1765 to 2012. Each simulation is checked for historical consistency to the range of simulations within the Climate Model Intercomparison Project phase 5 (CMIP5) ensemble. Only about 9×10^4^ simulations, from the initial 10-million simulations, are consistent with the CMIP5 historical ensemble. The model-parameter values for these simulations are then used to make future projections out to the year 2300.

Historic CO2 and radiative forcing is applied to the WASP model ensemble, starting in year 1765, followed by three future scenarios (Fig. 1): (1) RCP8.5 [2] representing unmitigated emission under business as usual (Fig. 1, red), (2) stabilisation at 2.0oC warming (Fig. 1, blue), and (3) stabilisation at 1.5oC warming (Fig. 1, grey), consistent with the Paris Agreement. The stabilisation scenarios follow RCP scenarios until the warming stabilisation target is crossed (RCP4.5 and RCP2.6 for the 2.0oC and 1.5oC stabilisation, respectively), and thereafter the annual carbon emissions are adjusted to maintain warming at the stabilisation target (Fig. 1a).

The thermosteric and ice-melt contributions to sea-level rise are simulated in WASP following Goodwin et al. (2017) [1] to year 2100. The thermosteric contribution is calculated from simulated ocean heat uptake after [3], while the ice-melt contribution is calculated using a semi-empirical approach [4] tuned to match the historic CMIP5 ensemble simulated ranges (see [1]). Beyond the year 2100, the ice-melt contribution per unit warming is assumed to slow over time with an exponential decay such that total ice-melt contribution to sea-level rise at equilibrium is equal to 2.3 m K-1 temperature anomaly [5]. The contribution to sea-level rise from changes in land-water storage is included up to the year 2100 (after [1]), using the projection ranges of [5] for land-water storage, and thereafter no land-water storage changes are considered to 2300 (Fig. 1b). The WASP model also projects the global-characteristic surface ocean pH over time for the three scenarios (Fig. 1c), calculating the change from the initial model pre-industrial surface ocean pH of 8.2 based on the Dissolved Inorganic Carbon increase in the surface ocean due to anthropogenic carbon uptake.

**The Dynamic Interactive Vulnerability Assessment (DIVA) Model**

DIVA is an integrated bio-geophysical coastal systems model driven by climate change and socio-economic development [6]. Climate change is represented by sea-level rise and other relevant climate factors such as storm characteristics are assumed to remain constant with time. Only the intersection of coastal topography and extreme sea levels, plus the modifying effect of defences, is considered when estimating flooding. Any erosion due to sea-level rise is not considered.

The world’s coast (excluding Antarctica) is represented by 12,148 linear coastal segments that have similar bio-physical and socio-ecological characteristics, and this is the basis for the analysis. For each coastal segment, global-mean sea-level rise derived from the WASP model as described above is downscaled to relative sea-level rise using estimates of segment-scale vertical land uplift or subsidence. These simulations consider vertical land movement due to isostatic effects [7] and enhanced subsidence in deltas to provide estimates of relative sea-level rise. Subsidence in 39 deltas was extracted from [8], with subsidence of 2mm/yr assumed as an average change in a further 77 deltas that are known to be subsiding. It is assumed that these vertical land movements continue at the same rate until 2300. Next, present extreme sea-level events (based on [9]) are adjusted by the magnitude of relative sea-level rise to determine flood levels over time. Hence, the land threatened by coastal flooding due to extreme sea levels can be defined assuming a planar water surface. The distribution of land elevation is derived from the Shuttle Radar Topographic Mission (SRTM) high resolution digital elevation model [10] and above 60°N and 60°S from the GTOPO30 dataset [11]. To calculate the people exposed to coastal flooding, the Global Rural Urban Mapping Project (GRUMPv1) dataset was employed [12, 13].

Future conditions are derived from sea-level rise scenarios (explained in the main text and under WASP in the supplementary material) and socio-economic scenarios which describe national changes in population and wealth. In this analysis, the five Shared Socioeconomic Pathways (SSPs) are considered [14, 15]. These reflect a range of potential and contrasting future population and economic conditions to 2100 and as a sensitivity analysis all of these scenarios are analysed. Population growth and decline assumes that changes follow the existing population distribution in each nation. Beyond 2100, a stable population is assumed for comparability following an agreement of the impact assessment community [16] and the 2100 population distribution is fixed to year 2300.

In addition, adaptation is explicitly considered in DIVA. In this analysis, adaptation is represented by protection in the form of sea dikes. These are estimated following a demand-for-safety function where safety (and dike height) increases with population density and wealth [17] (see also [6]). In this analysis, the dikes are initialised in 1995 and we assume no further upgrade so that the impacts (and any adaptation needs) due to SLR are apparent.

The output of the analysis is an estimate of risk – the expected annual population flooded. Given the assumptions made, especially the assumption of no adaptation and the very long time frame of the analysis, this parameter should be interpreted as an impact indicator rather than a projection of impacts. An increase in risk can be interpreted as an indicator of a demand for adaptation, as much as it can be interpreted as an indicator of impacts. Autonomous processes such as migration due to high flood frequency are not considered.

**Coastal City Defence Heights and Protected Population**

For the analysis, we extracted the following information from the database that was developed by [18] for the world’s 136 largest coastal cities: population distribution for different elevation levels in 2005, protection standards for all cities, and population growth scenarios by country for 2050 and 2100 from the Organisation for Economic Co-operation and Development (OECD). Country population scenarios were translated into city scenarios based on an extrapolation of the country’s urbanization rates. Following [18], it is assumed that city population growth has an upper limit of 35 million and in the absence of data we also assume that population and its distribution in 2300 will be the same as in 2100 (i.e., changes in dike heights and exposed population in 2300 compared to 2100 are only due to relative sea-level rise).

Protection standards that are expressed as return periods were translated to protection heights (expressed in meters above mean sea level) using the extreme sea-level data set developed by [9], which assumes that extreme events follow a Gumbel distribution. For adaptation during this century we assume that constant flood probability will be retained, which means that protection heights increase linearly with sea-level rise. For the long-range projection of 2300 it is assumed that all cities have upgraded to a protection standard equivalent to the 1,000-year still water level (i.e., events with 0.1% chance of occurrence in any given year).

We then use the relative sea-level rise scenarios, derived from the WASP model along with land uplift/subsidence rates (as described above under the DIVA model), and calculate the height of the flood defences, and the number of people across all 136 cities living in the protected flood plain.

**Relative sea-level rise analysis for deltas**

Fig. S1 shows the reduction in relative sea-level rise in hypothetical deltas to 2100 as a function of the sea-level rise scenarios calculated by the WASP model in Section 2 and plausible rates of subsidence as observed in deltas over the last 50 years [8, 19, 20]. The delta subsidence is assumed to continue linearly from 2000 to 2100. For the low bounding case, no subsidence, stabilisation at 2.0oC and 1.5oC avoids 33% and 47% of the relative rise, respectively. For the high bounding case, 10 mm/yr, the corresponding reduction in relative sea-level rise is only 14 to 19%, respectively. Intermediate cases of 3 mm/yr and 6 mm/yr are also presented.

<< Figure S1 about here >>

**Figure Captions**

Figure S1. Reduction in relative sea-level rise in a hypothetical subsiding delta from the years 2000 to 2100 given climate stabilisation at (a) 1.5°C and (b) 2.0°C relative to the RCP8.5 scenario. Contrasting plausible subsidence rates are considered from 0 to 10 mm/yr.

**Data**

The datasets supporting this article have been uploaded as part of the Supplementary Material.

**References**

1. Goodwin, P., Haigh, I. D., Rohling, E. J. & Slangen, A. 2017 A new approach to projecting 21st century sea-level changes and extremes. *Earths Future* **5**, 240-253. (DOI:10.1002/2016ef000508).
2. Meinshausen, M., Smith, S. J., Calvin, K., Daniel, J. S., Kainuma, M. L. T., Lamarque, J. F., Matsumoto, K., Montzka, S. A., Raper, S. C. B., Riahi, K., et al. 2011 The RCP greenhouse gas concentrations and their extensions from 1765 to 2300. *Climatic Change* **109**, 213-241. (DOI:10.1007/s10584-011-0156-z).
3. Williams, R. G., Goodwin, P., Ridgwell, A. & Woodworth, P. L. 2012 How warming and steric sea level rise relate to cumulative carbon emissions. *Geophysical Research Letters* **39**. (DOI:10.1029/2012gl052771).
4. Rahmstorf, S. 2007 A semi-empirical approach to projecting future sea-level rise. *Science* **315**, 368-370. (DOI:10.1126/science.1135456).
5. Church, J. A., P.U. Clark, A. Cazenave, J.M. Gregory, S. Jevrejeva, A. Levermann, M.A. Merrifield, G.A. Milne, R.S. Nerem, P.D. Nunn, et al. 2013 Sea Level Change. In *Climate Change 2013. The Physical Science Basis. Contribution of Working Group I to the Fifth Assessment Report of the Intergovernmental Panel on Climate Change* (eds. T. F. Stocker, D. Qin, G.-K. Plattner, M. Tignor, S. K. Allen, J. Boschung, A. Nauels, Y. Xia, V. Bex & P. M. Midgley). Cambridge, United Kingdom and New York, NY, USA, Cambridge University Press.
6. Hinkel, J., Lincke, D., Vafeidis, A. T., Perrette, M., Nicholls, R. J., Tol, R. S. J., Marzeion, B., Fettweis, X., Ionescu, C. & Levermann, A. 2014 Coastal flood damage and adaptation costs under 21st century sea-level rise. *Proceedings of the National Academy of Sciences of the United States of America* **111**, 3292-3297. (DOI:10.1073/pnas.1222469111).
7. Peltier, W. R. 2004 Global glacial isostasy and the surface of the ice-age earth: The ice-5G (VM2) model and grace. *Annual Review of Earth and Planetary Sciences* **32**, 111-149. (DOI:10.1146/annurev.earth.32.082503.144359).
8. Ericson, J. P., Vorosmarty, C. J., Dingman, S. L., Ward, L. G. & Meybeck, M. 2006 Effective sea-level rise and deltas: Causes of change and human dimension implications. *Global and Planetary Change* **50**, 63-82. (DOI:10.1016/j.gloplacha.2005.07.004).
9. Muis, S., Verlaan, M., Winsemius, H. C., Aerts, J. C. J. H. & Ward, P. J. 2016 Corrigendum: A global reanalysis of storm surges and extreme sea levels. *Nature Communications* **7**, 12913. (DOI:10.1038/ncomms12913).
10. Jarvis, A., Reuter, H. I., Nelson, A., & Guevara, E. 2008 Hole-filled SRTM for the globe Version 4. Available from the CGIAR-CSI SRTM 90m Database. [http://srtm.csi.cgiar.org](http://srtm.csi.cgiar.org/).
11. USGS. 2015 Global 30 Arc-Second Elevation (GTOPO30) dataset. (p. https://lta.cr.usgs.gov/GTOPO30.
12. Center for International Earth Science Information Network - Columbia University (CIESIN), International Food Policy Research Institute (IFPRI) & The World Bank and Centro Internacional de Agricultura Tropical (CIAT). 2011 Global Rural-Urban Mapping Project, Version 1 (GRUMPv1): Population Count Grid. (Palisades, NY, NASA Socioeconomic Data and Applications Center (SEDAC).
13. Balk, D. L., Deichmann, U., Yetman, G., Pozzi, F., Hay, S. I. & Nelson, A. 2006 Determining global population distribution: Methods, applications and data. In *Advances in Parasitology, Vol 62: Global Mapping of Infectious Diseases: Methods, Examples and Emerging Applications* (eds. S. I. Hay, A. Graham & D. J. Rogers), pp. 119-156.
14. Moss, R. H., Edmonds, J. A., Hibbard, K. A., Manning, M. R., Rose, S. K., van Vuuren, D. P., Carter, T. R., Emori, S., Kainuma, M., Kram, T., et al. 2010 The next generation of scenarios for climate change research and assessment. *Nature* **463**, 747-756. (DOI:10.1038/nature08823).
15. O'Neill, B. C., Kriegler, E., Riahi, K., Ebi, K. L., Hallegatte, S., Carter, T. R., Mathur, R. & van Vuuren, D. P. 2014 A new scenario framework for climate change research: the concept of shared socioeconomic pathways. *Climatic Change* **122**, 387-400. (DOI:10.1007/s10584-013-0905-2).
16. Frieler K. et al. (2017) Assessing the impacts of 1.5 °C global warming – simulation protocol of the Inter-Sectoral Impact Model Intercomparison Project (ISIMIP2b). *Geosci. Model Dev*., **10**, 4321-4345. https://doi.org/10.5194/gmd-10-4321-2017
17. Yohe G, Tol RSJ (2002) Indicators for social and economic coping capacity: Moving toward a working definition of adaptive capacity. *Glob Environ Change* **12(1)**:25–40.
18. Hallegatte, S., Green, C., Nicholls, R. J. & Corfee-Morlot, J. 2013 Future flood losses in major coastal cities. *Nature Climate Change* **3**, 802-806. (DOI:10.1038/nclimate1979).
19. Tessler, Z.D., Vorosmarty, C.J., Overeem, I., and Syvitski, J.P.M. 2017. A model of water and sediment balance as determinants of relative sea-level rise in contemporary and future deltas. *Geomorphology*. (Doi:10.1016/j.geomorph.2017.09.040)
20. Syvitski, J. P. M. 2008 Deltas at risk. Sustainability Science 3, 23-32. (DOI:10.1007/s11625-008-0043-3)
